# Development and Molecular Cytogenetic Identification of a Novel Wheat–*Leymus mollis* Lm#7Ns (7D) Disomic Substitution Line with Stripe Rust Resistance

**DOI:** 10.1371/journal.pone.0140227

**Published:** 2015-10-14

**Authors:** Xiaofei Yang, Changyou Wang, Xin Li, Chunhuan Chen, Zengrong Tian, Yajuan Wang, Wanquan Ji

**Affiliations:** College of Agronomy, State Key Laboratory of Crop Stress Biology for Arid Areas, Northwest A & F University, Yangling, Shaanxi, 712100, China; Institute of Genetics and Developmental Biology, CHINA

## Abstract

*Leymus mollis* (2*n* = 4*x* = 28, **NsNsXmXm**) possesses novel and important genes for resistance against multi-fungal diseases. The development of new wheat—*L*. *mollis* introgression lines is of great significance for wheat disease resistance breeding. M11003-3-1-15-8, a novel disomic substitution line of common wheat cv. 7182 –*L*. *mollis*, developed and selected from the BC_1_F_5_ progeny between wheat cv. 7182 and octoploid *Tritileymus* M47 (2*n* = 8*x* = 56, **AABBDDNsNs**), was characterized by morphological and cytogenetic identification, analysis of functional molecular markers, genomic *in situ* hybridization (GISH), sequential fluorescence *in situ* hybridization (FISH)—genomic *in situ* hybridization (GISH) and disease resistance evaluation. Cytological observations suggested that M11003-3-1-15-8 contained 42 chromosomes and formed 21 bivalents at meiotic metaphase I. The GISH investigations showed that line contained 40 wheat chromosomes and a pair of *L*. *mollis* chromosomes. EST-STS multiple loci markers and PLUG (PCR-based Landmark Unique Gene) markers confirmed that the introduced *L*. *mollis* chromosomes belonged to homoeologous group 7, it was designated as Lm#7**Ns**. While nulli-tetrasomic and sequential FISH-GISH analysis using the oligonucleotide Oligo-pSc119.2 and Oligo-pTa535 as probes revealed that the wheat 7**D** chromosomes were absent in M11003-3-1-15-8. Therefore, it was deduced that M11003-3-1-15-8 was a wheat–*L*. *mollis* Lm#7**Ns** (7**D**) disomic substitution line. Field disease resistance demonstrated that the introduced *L*. *mollis* chromosomes Lm#7**Ns** were responsible for the stripe rust resistance at the adult stage. Moreover, M11003-3-1-15-8 had a superior numbers of florets. The novel disomic substitution line M11003-3-1-15-8, could be exploited as an important genetic material in wheat resistance breeding programs and genetic resources.

## Introduction

*Leymus mollis* (Trin.) pilger (2*n* = 4*x* = 28, **NsNsXmXm**) is an important tetraploid species in *Leymus* (*Poaceae*: *Triticeae*) and a useful genetic resource for wheat breeding as a tertiary gene pool [1]. *L*. *mollis* has several desirable agronomic traits, such as a perennial growth habit, high fecundity, vigorous growth, strong rhizomes [2], especially good resistance to several fungal diseases, especially stripe rust, powdery mildew [3–5], as well as tolerance to alkaline and saline conditions, low temperatures, and drought stresses [6–9].

Stripe rust is one of the most destructive diseases of wheat resulting in serious yield losses in susceptible varieties, caused by the fungus *Puccinia striiformis* f.sp. *tritici* (*Pst*) [10,11]. Furthermore, due to rapid variation in the pathological races of stripe rust, it is really necessary to obtain and utilize new germplasms with resistance genes to breed resistant varieties [12,13]. Wide hybridization can transfer the desirable traits from wild relatives into common wheat and promote the new alien germplasms with advantageous exogenous genes [14], and introgression of alien resistance genes from wild relatives has been recognized as an efficient and environmentally safe approach to minimize yield losses due to diseases [15].

*Leymus mollis* has been extensively applied for hybridization with common wheat (*Triticum aestivum* L., 2*n* = 6*x* = 42, **AABBDD**) or durum wheat (*T*. *durum*, 2*n* = 4*x* = 28, **AABB**). In China, the first hybrids of common wheat 7182 and *L*. *mollis* were obtained by embryo rescue and colchicine treatment [16]. Later, many different derivatives have been developed, especially octoploid *Tritileymus* [17–20], which were extensively backcrossed with common wheat as a novel and useful bridge materials for wheat breeding. Several desirable characteristics, including those of drought tolerance, and high protein quality, especially the disease resistance, have been identified and offered valuable resources for wheat improvement. Osmotic-stress-responsive genes from *L*. *mollis* have also been incorporated into common wheat [8]. A translocation line Shannong0096 with resistance to stripe rust was developed from interspecific hybridization between common wheat cv. Yannong15 and octoploid *Tritileymus* M842 [17]. The multiple alien substitution line 05DM6 with resistance to stripe rust at the adult stage, which had three pairs of **Ns** chromosomes from *L*. *mollis*, was also selected from the progeny of octoploid *Tritileymus* M842-12 and *T*. *durum* cv. Trs-372 [20]. 10DM57, a 3**Ns** (3**D**) disomic substitution line with resistance to leaf rust was isolated from the F_5_ progeny of *T*. *durum* cv. D4286 × Octoploid *Tritileymus* M842-16 [18]. Previously, a wheat- *L*. *mollis* partial amphiploid M47 line with highly resistance to stripe rust and powdery mildew at the adult stage in field was developed [21]. In order to further transfer excellent resistance genes of M47 line into common wheat, the development of wheat- *L*. *mollis* chromosome addition line, substitution line or translocation line may be a novel step. And meanwhile, detecting and identifying alien chromosomes or segments in a wheat background is essential prior to their use in breeding programs [22–24]. Here, we characterized a novel wheat- *L*. *mollis* disomic substitution line via morphological observation, cytogenetic identification, functional molecular markers analysis, genomic *in situ* hybridization (GISH), sequential FISH-GISH and disease resistance evaluation.

Genomic *in situ* hybridization (GISH) and fluorescence *in situ* hybridization (FISH) are used routinely because they are the most efficient and accurate techniques to directly and precisely detect the alien chromosomes or introgression segments that introduced into the wheat backgrounds [24], and the constitution of chromosomes can thus be analyzed [25]. Moreover, diverse functional molecular markers are also powerful techniques, which can enable reliable identification of alien chromosomes or introgression fragments in wheat backgrounds, and can determine the homoeologous group relationships of alien chromosomes [15].

In the present study, we developed and identified a novel wheat—*L*. *mollis* Lm#7**Ns** (7**D**) disomic substitution line M11003-3-1-15-8, which was derived from the BC_1_F_5_ progeny of common wheat cv. 7182 and octoploid *Tritileymus* M47. It was revealed that the introduced *L*. *mollis* Lm#7**Ns** chromosomes contained new gene(s) for stripe rust resistance in wheat, which can be used as the starting material to incorporate the gene into the wheat genome through chromosome translocation.

## Materials and Methods

### Materials

The materials included wheat lines cv. 7182, Huixianhong (HXH), Chinese Spring (CS) (2*n* = 4*x* = 42, **AABBDD**), *L*. *mollis* (2*n* = 4*x* = 28, **NsNsXmXm**), octoploid *Tritileymus* M47 (2*n* = 8*x* = 56, **AABBDDNsNs**) and nulli-tetrasomics materials based on CS. A wheat—*L*. *mollis* disomic substitution line M11003-3-1-15-8, obtained from the BC_1_F_5_ progeny of common wheat cv. 7182 and octoploid *Tritileymus* M47, 7182 and *L*. *mollis* were the parents of M47, which were all maintained at the College of Agronomy, Northwest A&F University, China. 7182 was used as a control in electrophoretic analyses and in the agronomic trait evaluation. Huixianhong (HXH) was employed as the susceptible controls in the stripe rust resistance tests at the adult stage in the field.

## Methods

### Cytological identification

The roots and young spikes were all sampled in the field at their appropriate stage, respectively, and then the treatment of root tips and anthers were pretreated as described previously [21]. Cells with a complete chromosomes numbers of materials were photographed with an Olympus BX-43 microscope (Japan).

### GISH and FISH-GISH

The total genomic DNA of common wheat *L*. *mollis* and 7182 were isolated from seedling leaves using a modified CTAB method [26], with one additional purification step using chloroform to obtain high-quality DNA, which were used for GISH probes and blocks, respectively. The total genomic DNA of *L*. *mollis* was labeled with a Dig-Nick Translation Mix (Roche, Germany), sheared genomic DNA of 7182 was used as blocking DNA. The root tips were digested in2% cellulase and 1% pectinase at 37°C for 52–58 min (different digestion time should be needed in various materials); the slides and GISH procedure were performed as described previously [21]. Sequential FISH-GISH was performed to characterize the replaced wheat chromosomes. Oligonucleotide probes Oligo-pSc119.2 and Oligo-pTa535, 5’ end-labelled with 6- carboxyfluorescein (6-FAM) or 6-carboxy tetramethylrhodamine (Tamra) was synthesized by Shanghai Invitrogen Biotechnology Co.Ltd. (Shanghai, China), which were used for identifying the whole set of wheat chromosomes by sequential FISH-GISH analysis, probe labeling and *in situ* hybridization were performed according to Tang et al. [27]. Fluorescent signals were viewed and photographed (Olympus BX53, Japan) equipped with a Photometrics SenSys CCD camera DP 80.

### EST-STS and PLUG analysis

EST-STS markers (http://wheat.pw.usda.gov/SNP/new/pcr_primers.shtml) and PLUG (PCR-based Landmark Unique Gene) markers for homoeologous groups 1–7 of wheat chromosomes [13,28,29] were all synthesized in AuGCT DNA-SYN Biotechnology Co., Ltd of Beijing. These different markers were used to further determine homoeologous group relationships of the introduced alien chromosomes in the wheat—*L*. *mollis* disomic substitution line M11003-3-1-15-8. Polymerase chain reaction (PCR) assays were conducted as described previously [21]. The PCR products of EST-STS markers were separated in 8% non-denaturing polyacrylamide gel and visualized with silver staining, while the products of PLUG markers were analyzed by electrophoresis on a 1% agarose gel, to obtain high levels of polymorphism, an 7.5 μl aliquot of the product was digested with *Taq*I (65°C) for 2 h or *Hae*III (37°C) for 2 h, respectively. Digested fragments were fractionated by electrophoresis on 2% agarose gel in TAE buffer.

### Disease resistance and agronomic trait evaluation

To evaluate resistance to stripe rust at the adult stage, a mixture of *Puccinia striiformis* f. sp. *tritici* (*Pst*) races was used, including CYR31, CYR32, CYR33 and Shuiyuan 11 from Shaanxi Province. Common wheat cv. 7182, *L*. *mollis*, line M11003-3-1-15-8, and the susceptible control variety HXH was separately tested in the field at the College of Agronomy, Northwest A & F University.

When the control variety HXH were all fully infected after the artificial inoculation, the reactions to the mixed *Pst* races were scored according to a previously published method, the infection types (IT) scores of wheat stripe rust at adult stage was assessed on a scale from 0–4, as follows: 0, 0; and 1 were considered to be resistant, 2 was recorded to be moderately resistant, 3 and 4 was assessed to be moderately susceptible and susceptible, respectively [30].

Morphological traits of line M11003-3-1-15-8 and its parents, common wheat cv. 7182, *L*. *mollis*, M47, i.e., plant height, plant type, spike length, spikelets per spike, kernels per spikelet, kernels per spike, thousand kernel weight, awnless, self-fertility and maturity, were all sampled randomly and investigated.

## Results

### Morphology and cytological characterization

**Fig 1:**
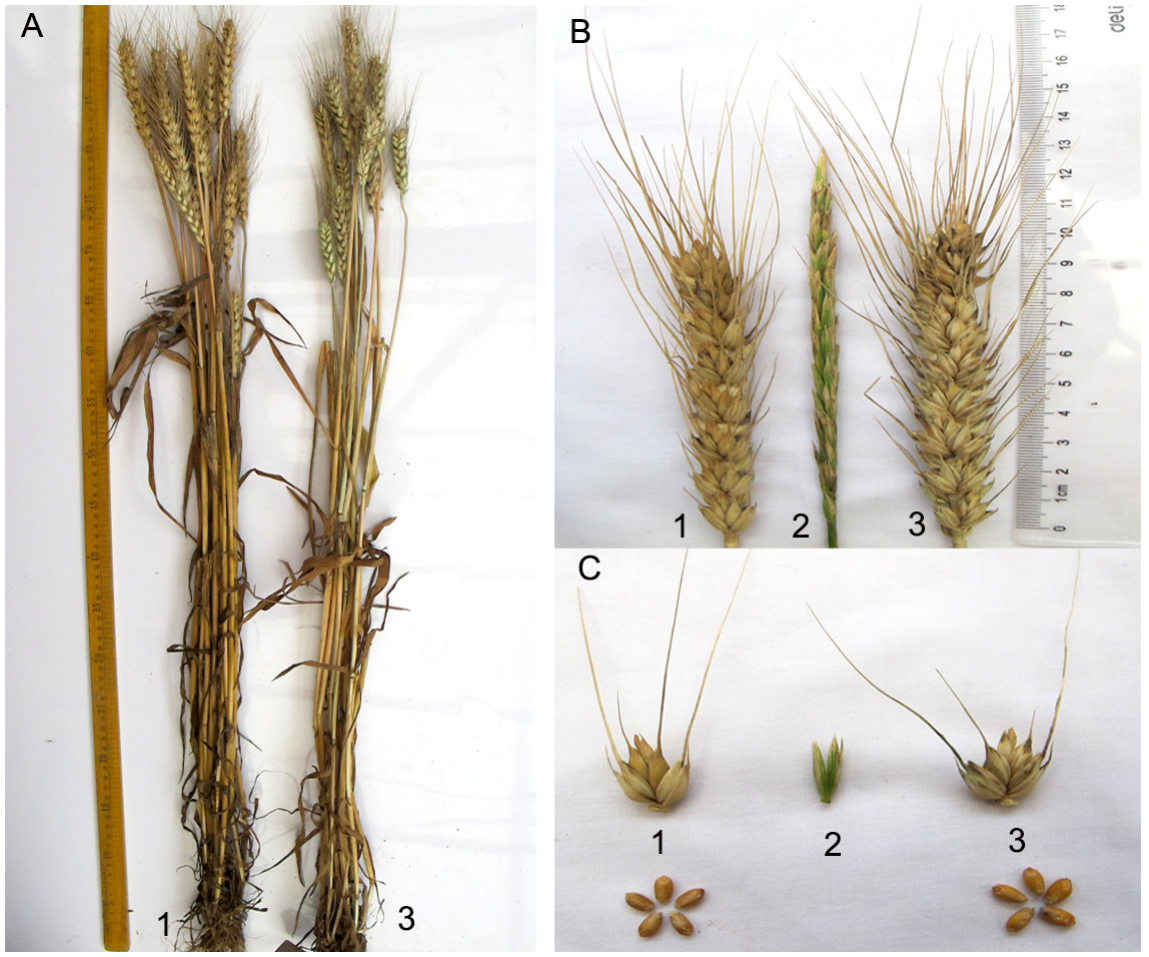
Morphological comparison of adult plants, spikes, spikelets, and seeds from wheat—*L*. *mollis* derivative line M11003-3-1-15-8 and its parents, common wheat cv. 7182 and *L*. *mollis*. a adult plants, b spikes, c spikelets and seeds. 1 7182, 2 M11003-3-1-15-8, 3 *Leymus mollis*.

**Fig 2:**
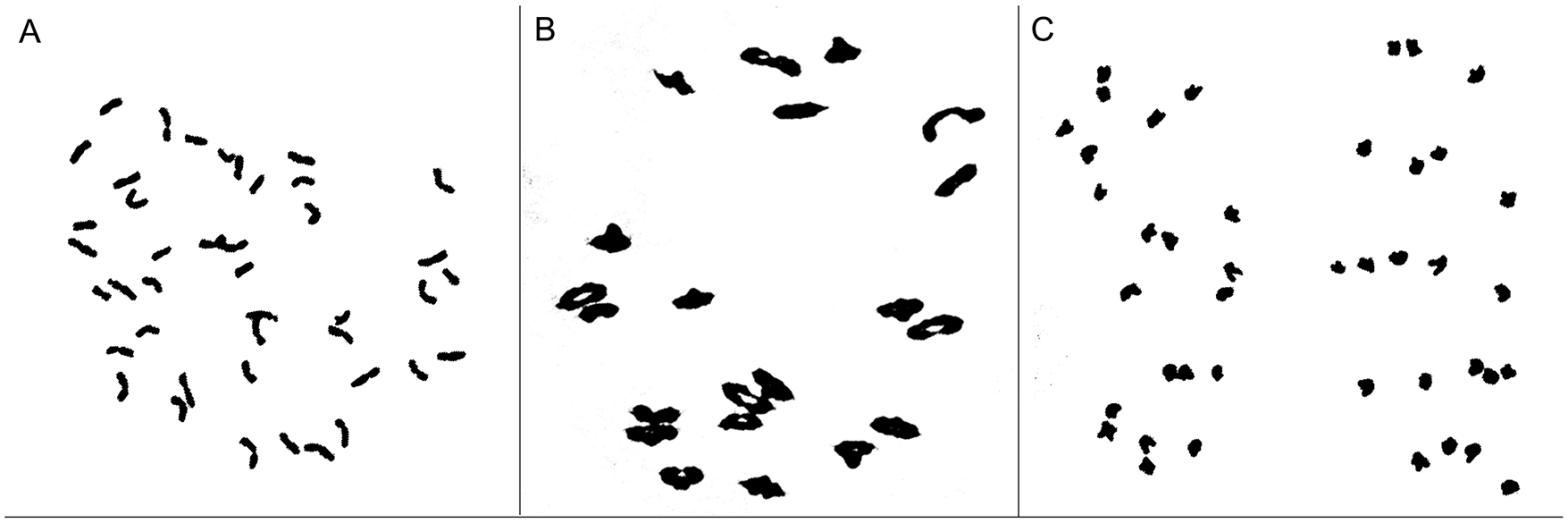
Mitotic (a), meiotic I (b) and anaphase I (c) chromosome characteristics of M11003-3-1-15-8, a 2*n* = 42, b 2*n* = 21П, c 2*n* = 21+21.

M11003-3-1-15-8 was derived from BC_1_F_5_ progenies of wheat cv. 7182 and partial amphiploid M47 (2*n* = 8*x* = 56, **AABBDDNsNs**). Plant height, plant type, spike length, long awns of M11003-3-1-15-8 closely resembled those of wheat parent 7182, and exhibiting high seed set as wheat cv. 7182. But M11003-3-1-15-8 had greater kernels per spikelet, higher thousand kernel weight and later maturity than 7182, which were significantly different from wheat parent 7182. No seeds were produced by self- and cross-pollination in *L*. *mollis*. (Table 1, Fig 1). The mitotic and meiotic observations of line M11003-3-1-15-8 indicated that root tip cells (RTCs) had a chromosome number of 42 (Fig 2a), pollen mother cells (PMCs) formed a pairing configuration of 21 II (Fig 2b), and the average chromosome configuration at MI was 0.17 univalent, 1.21 rod, 19.62 ring bivalents, no trivalents or quadrivalents were observed at metaphase I, meanwhile no chromosomes was lagged at anaphase I (Table 2, Fig 2c). Therefore, line M11003-3-1-15-8 exhibited highly cytological stability.

**Table 1:** Agronomic traits of wheat parent cv. 7182, *L*. *mollis*, M47 and disomic substitution line M11003-3-1-15-8.

Materials	Plant height(cm)	Plant type	Spike length(cm)	Spikelets/ spike	Florets/spikelet	Kernels/spike	Thousand kernel weight (g)	Awnedness
7182	86±3	Tighten	10.0±0.4	20±2	4±1	46±4	37.3 ±0.5	long awn
*L*. *mollis*	56±2	Drooping	14.7±0.3	20±2	2±1	1±1	-	awnless
M11003-3-1-15-8	83±2	Tighten	10.5±0.5	22±2	5±1	49±6	41.5 ± 0.4**	long awn

**indicates significant differences between the substitution line M11003-3-1-15-8 and wheat parent 7182 (P < 0.01).

- represent no data.

**Table 2:** Chromosome numbers and configuration pairing at mitotic and meiotic of M11003-3-1-15-8.

Material	2n	No. of cells observed	Chromosome configuration					
			Univalent	Bivalent			Trivalent	Quadrivalent
				Rod	Ring	Total		
M11003-3-1-15-8	42	77	0.17(0–2)	1.21(1–3)	19.62(19–21)	20.83(20–21)	0	0

### Molecular markers analysis

**Fig 3:**
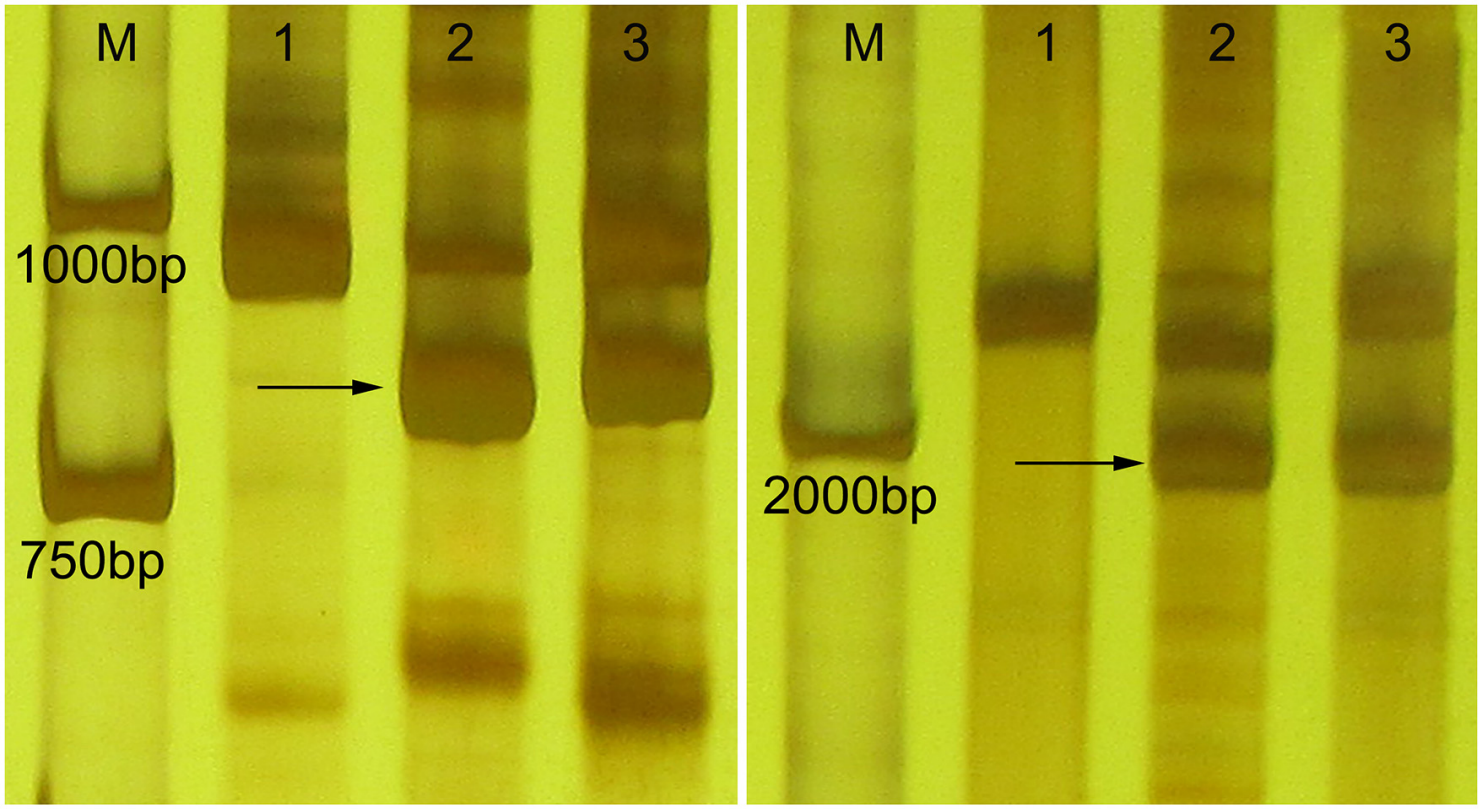
EST-STS markers results of M11003-3-1-15-8. The arrow indicates an *L*. *mollis* specific band. M DL2000, 1 7182, 2 *L*.*mollis*, 3 M11003-3-1-15-8. *BE482781-*7**A**L 7**B**L 7**D**L, *BE404955-*7**A** 7**B**S 7**D**.

**Fig 4:**
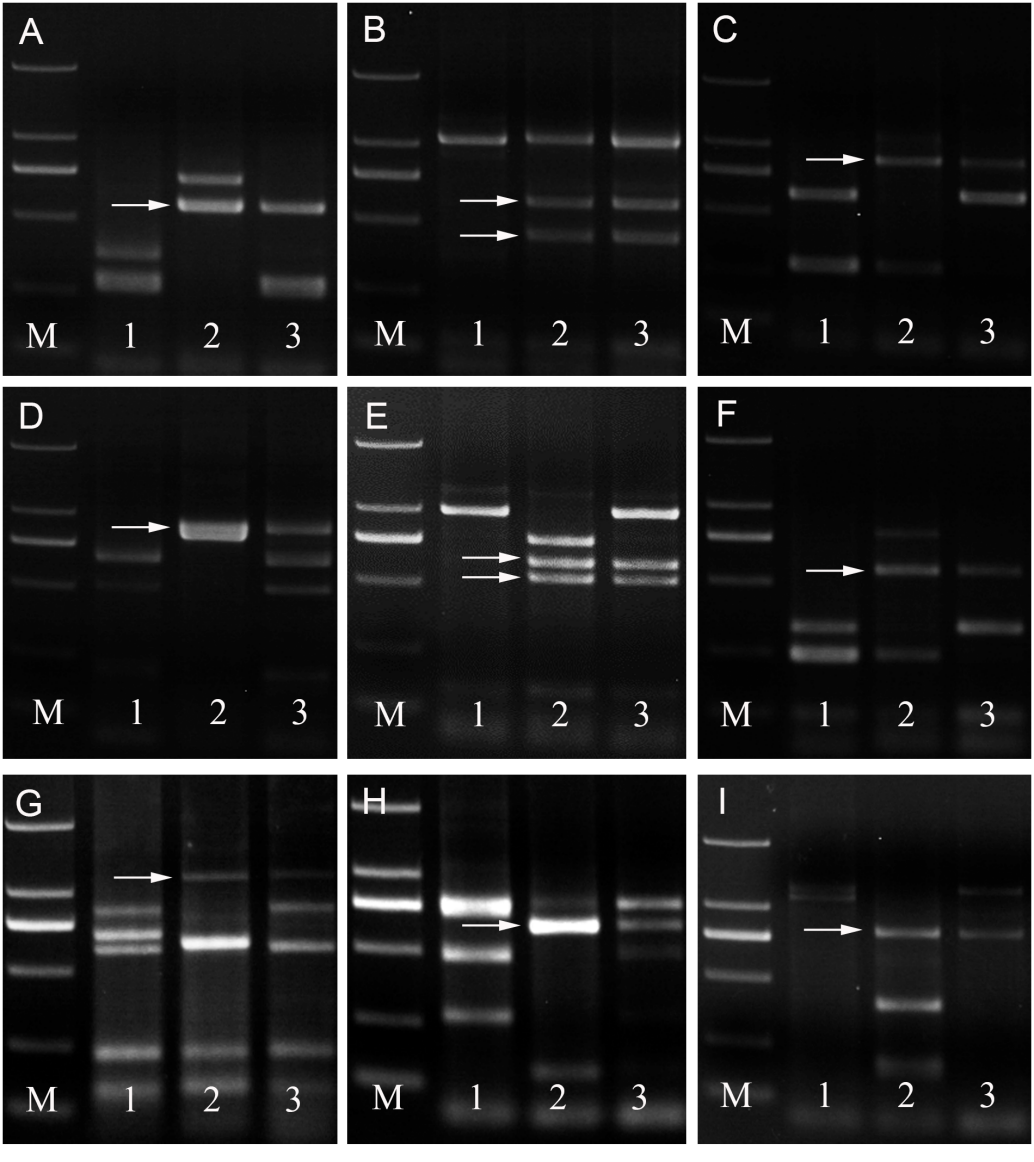
PLUG markers results of M11003-3-1-15-8. The arrow indicates an *L*. *mollis* specific band. All PCR products are digested by *Taq*I or *Hae*III. M DL2000, 1 7182, 2 *L*.*mollis*, 3 M11003-3-1-15-8. a TNAC1811-*Hae*III, b TNAC1812-*Hae*III, c TNAC1926-*Hae*III, d TNAC1956-*Hae*III, e TNAC1812-*Taq*I, f TNAC1926-*Taq*I, g TNAC1826-*Taq*I, h TNAC1903-*Taq*I, i TNAC1957-*Taq*I.

Molecular markers were used to determine the homoeologous group relationships of the alien chromosomes. In the present study, two EST-STS markers and seven PLUG markers, i.e., *BE482781-*7**A**L 7**B**L 7**D**L, *BE404955-*7**A**L 7**B**L 7**D**L, TNAC1811–*Hae*Ш, TNAC1812-*Hae*Ш */ Taq*І, TNAC1926-*Hae*Ш */ Taq*l, TNAC1956-*Hae*Ш, TNAC1826-*Taq*l, TNAC1903-*Taq*l, TNAC1957-*Taq*l which mapped onto the seventh homoeologous group, amplified clear polymorphic bands in M11003-3-1-15-8 and *L*. *mollis* but not in the wheat parent cv. 7182. It was suggested that these markers could be used as specific markers of *L*. *mollis* chromosomes in M11003-3-1-15-8 (Table 3, Figs 3 and 4), and meanwhile the introduced pair of *L*. *mollis* chromosomes in M11003-3-1-15-8 was demonstrated that they were homoeologous with the seventh homoeologous group chromosomes of wheat.

**Table 3:** EST-STS and PLUG polymorphic markers mapped on seventh homoeologous groups applied to linkage analysis of the Lm#7Ns.

**marker**	Primer(5’-3’)	location	gel type/restriction enzyme	Tm(°C)/t (h)
*BF482781*	F: CATCAGGAAGTCTAAGGCCG R: GAGAAGCAACCCAGCAACTC	7**A**L7**B**L 7**D**L	8% non-denaturing polyacrylamide gel / -	60 / -
*BE404955*	F: CGTGGCATTATAGCGAGGAT R: ATTGGTGAAGCAGAAGCGAT	7**A**L 7**B**L 7**D**L	8% non-denaturing polyacrylamide gel / -	60 / -
*TNAC1811*	F: CTGCTCAACGAGTTCATCGAC R: TTGGAGTGGACGTTGCATT	7**A**L7**B**L 7**D**L	2% agarose gel / TaqI	60 / 2
*TNAC1812*	F: ACTTCGCTTGGTCTCCTCAAT R: GAGAAGTGTGCCAATTCCAAA	7**A**L7**B**L 7**D**L	2% agarose gel /TaqI/HaeIII	60 / 2 or 3
*TNAC1926*	F: CGTCAGCTACAGCGACATCTA R: AACTTGAGCAGCGTGGTGTT	7**A**L7**B**L 7**D**L	2% agarose gel /TaqI/HaeIII	60 / 2 or 3
*TNAC1956*	F: ACGAAGGACAATTGCTGCTAA R: GTGCACTTCTTGCCCTACTTG	7AL7BL 7DL	2% agarose gel / TaqI	60 / 2
*TNAC1826*	F: CACATATGATGATGACGGCAAT R: GGCAGGGAGGAAACTCTACTG	7**A**L7**B**L 7**D**L	2% agarose gel / TaqI	60 / 2
*TNAC1903*	F: TCGCTTCTTCTGCTTGTTCTT R: CTGCTACTAGGCCACCCAAA	7**A**L7**B**L 7**D**L	2% agarose gel / TaqI	60 / 2
*TNAC1957*	F: TCAACATTTGCAGGATTGTCA R: TTTCACAGGAACCTCTGCATC	**7AL7BL** 7**D**L	2% agarose gel	60 / 2

- represent no data.

### Nulli-tetrasomic analysis

**Fig 5:**
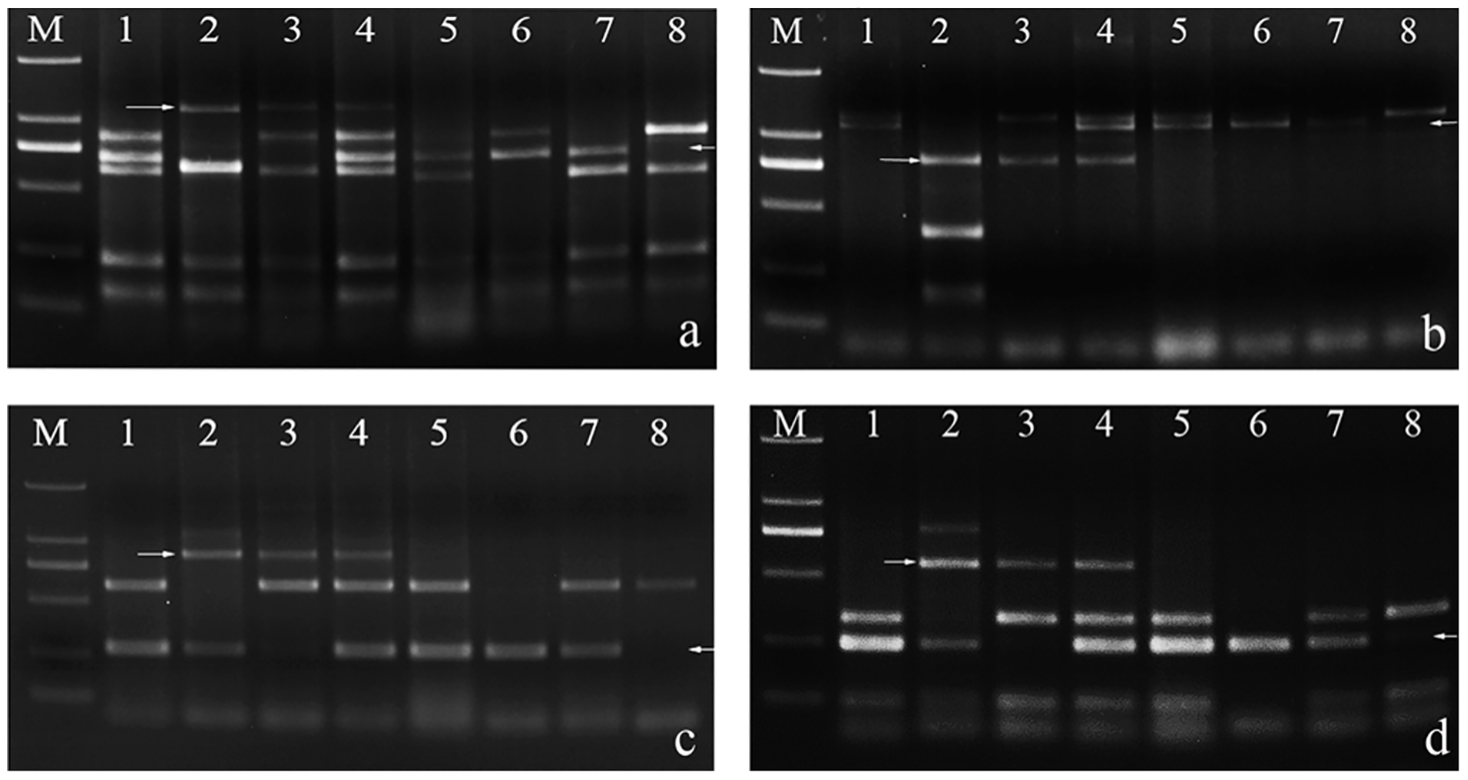
Nullisomic-tetrasomic analysis of M11003-3-1-15-8. The arrow indicates an *L*. *mollis* specific band. M DL2000, 1 7182, 2 *L*.*mollis*, 3 M11003-3-1-15-8, 4 M47, 5 CS, 6 CSN7AT7D, 7 CSN7BT7A, 8 CSN7DT7B; a TNAC1826-*Taq*I, b TNAC1957-*Taq*I, c TNAC1926-*Hae*III, d TNAC1926-*Taq*I.

Based on the amplification of nulli-tetrasomic materials of wheat Chinese Spring (CS), the seventh homoeologous group PLUG markers can amplify diagnostic bands in wheat chromosomes 7**A**, 7**B**, and 7**D** specific bands, respectively. As shown in Fig 5, three PLUG primers TNAC1826-*Taq*l, TNAC1957-*Taq*l and TNAC1926-*Hae*Ш */ Taq*l clearly amplified fragments of chromosomes 7**A**, 7**B**, and 7**D** in CS, the *L*. *mollis* -specific bands appeared in M11003-3-1-15-8, it was also suggested that the introduced *L*. *mollis* chromosome in M11003-3-1-15-8 belonged to homoeologous group 7, while the chromosome 7**D**-specific band in M11003-3-1-15-8 was significantly absent, therefore it was preliminarily deduced that wheat chromosomes 7**D** were absent in M11003-3-1-15-8.

### GISH and FISH-GISH analysis

**Fig 6:**
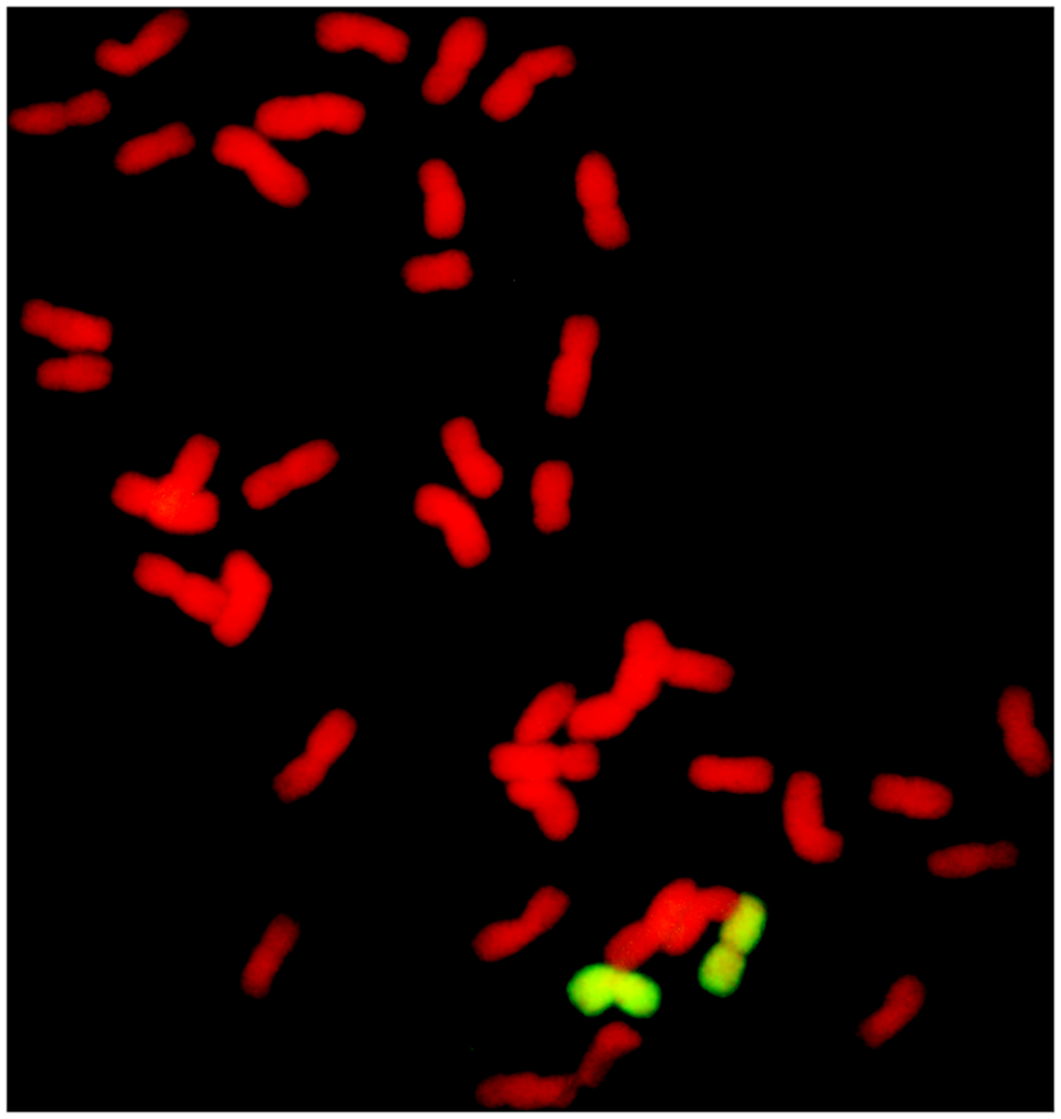
Genomic *in situ* hybridization (GISH) analysis using *L*. *mollis* genomic DNA (green) as probe on root tip metaphase chromosomes of M11003-3-1-15-8. Chromosomes were counterstained with PI (red).

For GISH, the total genomic DNA of wheat cv. 7182 and *L*. *mollis* were used as the block and probe respectively, to identify the introduced *L*. *mollis* chromosomes in M11003-3-1-15-8. GISH results of somatic cells showed that M11003-3-1-15-8 had two alien chromosomes with clear hybridization signals (Fig 6).

**Fig 7:**
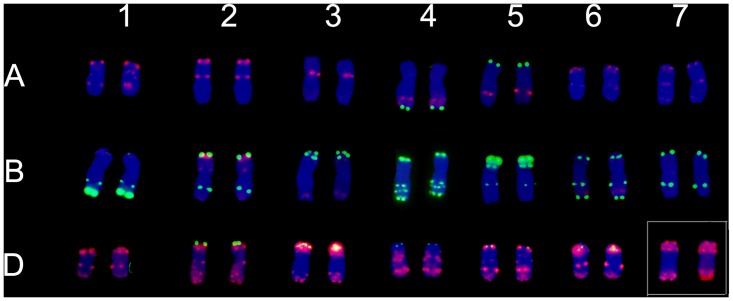
Fluorescence *in situ* hybridization (FISH) analysis using Oligo-pTa535 (red), Oligo-pSc119.2 (green) as probes on root tip metaphase chromosomes of 7182. Chromosomes were counterstained with DAPI (blue). The pair of wheat chromosomes 7**D** was indicated in a small white rectangular box.

**Fig 8:**
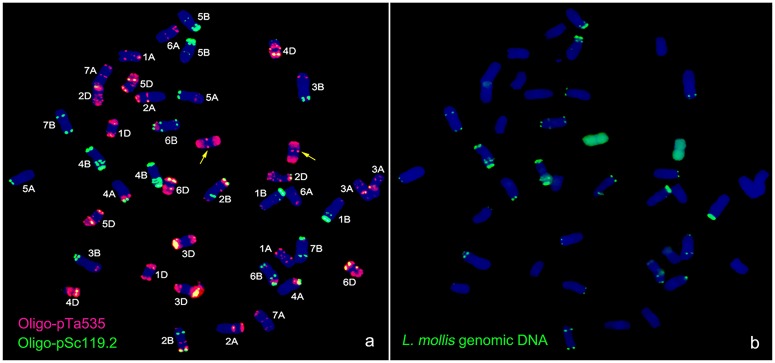
Fluorescence *in situ* hybridization (FISH) analysis using Oligo-pTa535 (red), Oligo-pSc119.2 as probes on root tip metaphase chromosomes of M11003-3-1-15-8 (a), Genomic *in situ* hybridization (GISH) analysis using *L*. *mollis* genomic DNA (green) as probe on root tip metaphase chromosomes of M11003-3-1-15-8. Chromosomes were counterstained with DAPI (blue). The yellow arrows indicate the introduced *L*. *mollis* chromosomes in M11003-3-1-15-8.

In order to further determine the wheat chromosomes replaced by *L*. *mollis* chromosomes in M11003-3-1-15-8 line, sequential FISH-GISH analysis was performed. Oligo-pTa535, mainly detecting wheat **D**-genome chromosomes, and Oligo-pSc119.2, mainly identifying **B**-genome chromosomes, which can successfully discriminate the whole set of common wheat 42 chromosomes by combining the two oligonucleotide probes. Therefore, first, the standard FISH karyotype patterns of wheat parent cv. 7182 (Fig 7) was painted using probes Oligo-pTa535 and Oligo-pSc119.2 according to Tang et al. (2014). And also, these two probes were used to hybridize the M11003-3-1-15-8 mitotic spread chromosomes by mutil-color FISH-GISH (Fig 8). Compared with the standard FISH painting of wheat parent cv. 7182, it was deduced that the pair of wheat chromosomes 7**D** was really absent in M11003-3-1-15-8, which was consistent with the results of nulli-tetrasomic analysis. Namely, the wheat chromosomes 7**D** were replaced by *L*. *mollis* chromosomes Lm#7**Ns**

### Disease resistance evaluation

**Fig 9:**
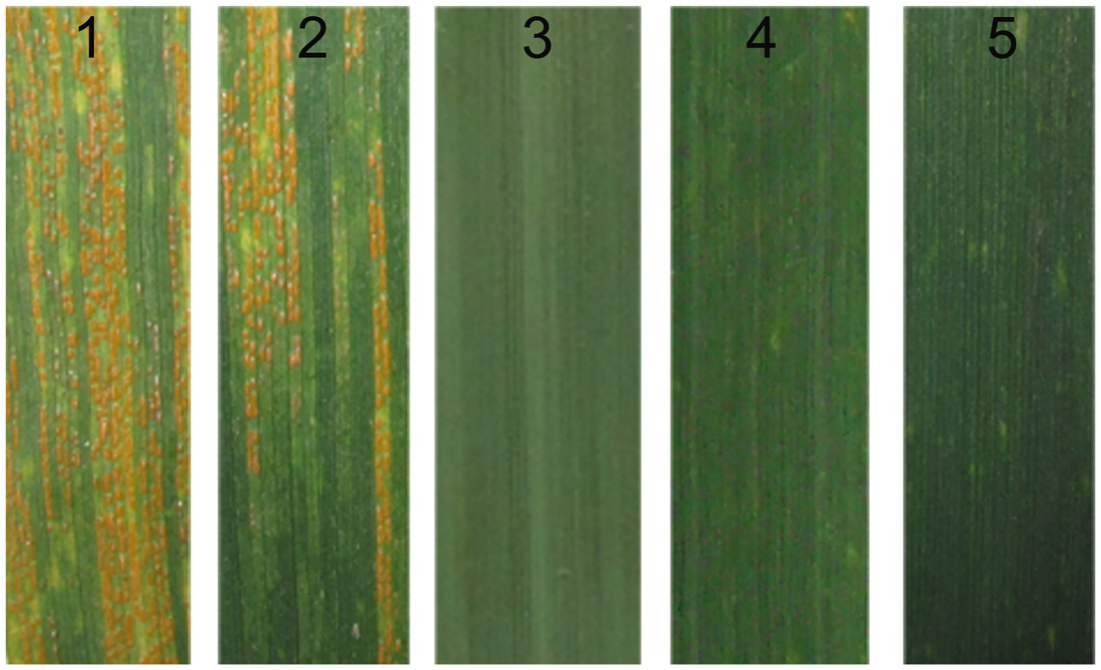
Infection type for *Pst* of susceptible control variety Huixianhong (HXH) (1), 7182 (2), *L*. *mollis* (3), octoploid *Tritileymus* M47 (4) and M11003-3-1-15-8 (5) at the adult stage. A score of wheat stripe rust at adult stage: resistant: 0–2, susceptible: 3.

The reactions of adult plants to the mixed *Pst* races (CYR30, CYR31, CYR32 and Shuiyuan11) were tested in the field. Wheat parent cv. 7182 and control variety HXH were susceptible to stripe rust when infected with a mixture of the corresponding physiological races, *L*. *mollis* was almost immune to these isolates whereas, and M11003-3-1-15-8 also performed highly resistance to stripe rust at the adult stage (Table 4, Fig 9). The results indicated that the stripe rust resistance of M11003-3-1-15-8 was inherited from the M47 parent, and was traced to *L*. *mollis*.

**Table 4:** Evaluation of the disease resistance to powdery mildew and stripe rust of 7182, *L*. *mollis*, M47 and M11003-3-1-15-8.

Material	RTC	PMC	Alien chromosome type	Stripe rust resistance
7182	42	21 II	-	3
*L*.*mollis*	28	-	**NsNsXmXm**	**0**
M47	56	28 II	**AABBDDNsNs**	**0;**
M11003-3-1-15-8	42	21 II	Lm#7**Ns** (7**D**)	**1**
HXH	42	21 II	-	4

A score of wheat stripe rust at adult stage: resistant: 0–2, susceptible: 3–4.

- represent no data

## Discussion

*Leymus mollis* could perform good resistance to fungal diseases such as powdery mildew, stripe rust, leaf rust and scab [3–5]. In previous study, various wheat- *L*. *mollis* derivatives with alien chromosomes distributed in different homoeologous groups were developed, especially octoploid *Tritileymus* derived from wheat cv. 7182 and *L*. *mollis* could be used as an excellent intermediate resistant material for wheat disease resistance breeding, which incorporated a novel stripe rust and powdery mildew resistance genes from *L*. *mollis* [17–21,31].

In the present study, M11003-3-1-15-8, a new Lm#7**Ns** (7**D**) substitution line with high resistance to stripe rust carried one **Ns** chromosome pair derived from *L*. *mollis* was further developed and screened. It could thus be an interesting germplasm for disease resistance breeding.

The stable heritability of alien chromosomes is theoretically and practically significant, therefore, the chromosome constitution and configuration pairing in RTCs and PMCs of M11003-3-1-15-8 was identified by cytological observations, respectively. The results suggested that M11003-3-1-15-8 exhibited highly cytological stability based on 21 bivalents during meiotic metaphase I and no lagging chromosomes at metaphase I. GISH is the visible, accurate and most essential technique for tracking alien chromosomes introduced into the wheat background. To detect the alien chromosomes in M11003-3-1-15-8, GISH was performed using *L*. *mollis* and wheat parent cv. 7182 as probe and block respectively at the ratio of 1: 20, it was clearly detected two alien chromosomes with yellow-green hybridization signals in M11003-3-1-15-8. In a word, M11003-3-1-15-8 was cytogenetically stable and carried one pair of *L*. *mollis* chromosomes.

To further characterize M11003-3-1-15-8 line, sequential FISH-GISH and nulli-tetrasomic analysis were carried out to determine the substituted wheat chromosomes in M11003-3-1-15-8. FISH is a powerful technique for localizing highly repetitive DNA sequences, detecting specific sites in particular regions of individual chromosomes and discriminate genome constitutions [32,33]. Many substitution lines related to common wheat chromosomes and alien chromosomes have been identified using FISH or nulli-tetrasomic analysis, including 6**G**(6**B**) [34], **1St(**1**D**) [28], 6**Js**/6**B** [13], 2**R** ^**a**^(2**D**) [35], 2**Ns**(2**D**) [36], 2**V** ^**b**^(2**D**) [37], and 3**D**(3**Ns**) [18]. In this study, the standard FISH karyotype patterns of wheat parent 7182 was painted for the first time using oligonucleotide probes Oligo-pTa535 and Oligo-pSc119.2, which was convenient to distinguish common wheat chromosomes in M11003-3-1-15-8. Thus, based on the sequential FISH-GISH and nulli-tetrasomic analysis, it was deduced that the pair of chromosomes 7**D** was significantly absent in M11003-3-1-15-8.

Molecular markers could provide a simple and precise approach to track the alien DNA in a wheat background based on comparative genome analysis, and could determine the homoeologous group relationship of alien chromosomes in a wheat background, especially those from the conserved genetic region showing high levels of collinearity among the cereal genomes, such as rice, Brachypodium, and wheat [38]. EST-SSR and EST-STS markers could be used to distinguish alien chromosome homoeologous group relationship between aliens chromosomes and those of wheat, and track alien chromosomes [39]. More recently, EST–PCR markers have been employed extensively in terms of convenient manipulation and application as effective tools for genetic and homoeologous relationship analysis of *Agropyron cristatum* [40], *Dasypyrum villosum* [41], *Hordeum californicum* [39], *Hordeum chilense* [42], *Secale cereale* L. [43], *Thinopyrum intermedium* [28], *Thinopyrum ponticum* [44], *Secale africanum* [35], *Psathyrostachys huashanica* [45]. In addition, PLUG markers could not only amplify polymorphism among wheat **A**, **B** and **D** genomes due to the intron polymorphisms, displayed similar homoeologous chromosome locations to hexaploid wheat [29,46], collinear gene relationships among *Triticeae* species and sequence polymorphism among different species suggested that PLUG markers could also be used to determine homoeologous group relationships of alien chromosomes. Hu et al. revealed genomic rearrangement between wheat and *Th*. *elongatum* using mapped functional molecular markers (EST-SST and PLUG markers). In the present study, we used EST–STS multiple-loci pair primers and PLUG molecular markers from the seven homoeologous groups of wheat to further determine the homoeologous relationships between the introduced *L*. *mollis* chromosome and those of common wheat. Two EST–STS markers and seven PLUG markers located on wheat chromosome group 7 could specifically amplify polymorphism bands in M11003-3-1-15-8 and *L*. *mollis*, it was suggested that the pair of *L*. *mollis* chromosomes in M11003-3-1-15-8 belonged to homoeologous group 7. And meanwhile, these special markers could also be used as unique tools for tracking alien *L*. *mollis* in a wheat background, and for comparative gene mapping, chromosomal evolutionary analysis, and gene introgression during wheat improvement using *L*. *mollis* accessions as gene donors.

In summary, we further developed a new wheat- *L*. *mollis* disomic substituion line M11003-3-1-15-8 that was produced spontaneously from the BC_1_F_5_ progeny of a cross between octoploid *Tritileymus* M47 and wheat parent 7182. The introduced *L*. *mollis* chromosomes in M11003-3-1-15-8 were associated with homoeologous group 7 of wheat chromosome by cytogenetic, FISH-GISH, and functional molecular markers analysis, and it was temporarily designated as Lm#7**Ns**. While FISH-GISH and nulli-tetrasomic analysis results demonstrated that the pair of wheat chromosomes 7**D** were absent in M11003-3-1-15-8. Therefore, M11003-3-1-15-8 is a new Lm#7**Ns** (7**D**) substitution line. The self-fertility of M11003-3-1-15-8 was as high as 91.0%, which approached the normal level found in common wheat, suggested that all of the chromosomes in M11003-3-1-15-8 had normal pairing and separation. Furthermore, although wheat group 7 chromosomes contained the spike characteristics such as spike length and grain weight genes [45], M11003-3-1-15-8 did not take along negative spike traits, this also indicated that the Lm#7**Ns** chromosome from *L*. *mollis* could compensate for the absence of the 7**D** chromosome from wheat. What’s more, wheat—*L*. *mollis* disomic substitution line M11003-3-1-15-8 was highly resistant to stripe rust at adult stage, it was revealed that the *L*. *mollis* chromosome Lm#7**Ns** possessed stripe rust resistances gene(s). Moreover, the superior spike trait of substitution line M11003-3-1-15-8 will be particularly significant for enhancing the crop yield because it allows more kernels to be formed per spike. It is worth noting that several *L*. *mollis* chromosomes contain novel stripe rust resistance genes, and the introgressed genes can be expressed in the wheat backgrounds.

In a word, M11003-3-1-15-8 was a novel wheat–*L*. *mollis* Lm#7**Ns** (7**D**) disomic substitution line with stripe rust resistance, which could be exploited as an important genetic material in wheat resistance breeding programs and genetic resources.

## References

[1] Kishii M, Wang RC, Tsujimoto H (2003) Characteristics and behaviour of the chromosomes of *Leymus mollis* and *L. racemosus* (Triticeae, Poaceae) during mitosis and meiosis. Chromosome Res 11: 741–748. 14712860 10.1023/b:chro.0000005774.00726.71

[2] Sigurbjornsson B (1960) Studies on the Icelandic *Elymus*. PhD thesis Cornell University, Ithaca, New York.

[3] Fatih A (1983) Analysis of the breeding potential of wheat-*Agropyron* and wheat-*Elymus* derivatives, 1 Agronomic and quality characteristics. Hereditas 98: 287–295. 6874401 10.1111/j.1601-5223.1983.tb00607.x

[4] Merker A (1992) The Triticeae in cereal breeding. Hereditas 116: 277–280.

[5] Mujeeb-Kazi A, Bernard M, Bekele GT, Mirand JL (1983) Incorporation of alien genetic information from *Elymus* giganteus into Triticum aestivum. In: Sakamoto S (ed) Proceedings of the 6th international wheat genetics symposium Plant germplasm Institute, Kyoto, Japan,: 223–231.

[6] Anamthawat-Jónsson K, Bödvarsdóttir SK, Bragason BT, Gudmundsson J, Martin PK, Koebner RMD (1997) Wide hybridization between wheat (*Triticum* L.) and lymegrass (*Leymus* Hochst.). Euphytica 93: 293–300.

[7] Habora ME, Eltayeb AE, Oka M, Tsujimoto H, Tanaka K (2013) Cloning of allene oxide cyclase gene from *Leymus mollis* and analysis of its expression in wheat-*Leymus* chromosome addition lines. Breed Sci 63: 68–76. 10.1270/jsbbs.63.68 23641183 PMC3621447

[8] Habora ME, Eltayeb AE, Tsujimoto H, Tanaka K (2012) Identification of osmotic stress-responsive genes from *Leymus mollis*, a wild relative of wheat (*Triticum aestivum* L.). Breed Sci 62: 78–86. 10.1270/jsbbs.62.78 23136517 PMC3405956

[9] McGuire PE, Dvorak J (1981) High salt-tolerance potential in wheatgrass. Crop science 21: 702–705.

[10] Chen XM, Penman L, Wan AM, Cheng P (2010) Virulence races of Puccinia *striiformisf*. sp.*triticiin* 2006 and 2007 and development of wheat stripe rust and distributions, dynamics, and evolutionary relationships of races from 2000 to 2007 in the United States. Can J of Plant Pathol 32: 315–333.

[11] Yaniv E, Raats D, Ronin Y, Korol AB, Grama A, Bihamta MR, et al (2015) Evaluation of marker-assisted selection for the stripe rust resistance gene Yr15, introgressed from wild emmer wheat. Mol Breeding 35: 1–12. 10.1007/s11032-015-0238-0 PMC5091809 27818611

[12] Du WL, Wang J, Lu M, Sun SG, Chen XH, Zhao JX, et al (2014) Characterization of a wheat-*Psathyrostachys* huashanica Keng 4Ns disomic addition line for enhanced tiller numbers and stripe rust resistance. Planta 239: 97–105. 10.1007/s00425-013-1957-2 24085532

[13] Hu LJ, Li GR, Zeng ZX, Chang ZJ, Liu C, Yang ZJ (2011) Molecular characterization of a wheat -*Thinopyrum ponticum* partial amphiploid and its derived substitution line for resistance to stripe rust. J Appl Genet 52: 279–285. 10.1007/s13353-011-0038-0 21437653

[14] Anamthawat-Jónsson K (1995) Wide-hybrids between wheat and *lymegrass*: breeding and agricultural potential. Icel Agr Sci 9: 101–113.

[15] Niu ZX, Klindworth DL, Wang RRC, Jauhar PP, Larkin PJ, Xu SS (2011) Characterization of HMW glutenin subunits in *Thinopyrum intermedium, Th. bessarabicum, Lophopyrum elongatum, Aegilops markgrafii*, and their addition lines in wheat. Crop Sci 51: 667–677.

[16] Chen SY, Fu J, Gao LZ (1985) The hybridization between *Triticum* aestivum and *Leymus mollis* . Acta Botanica Boreali-Occidentalia Sinica 4: 260–266.

[17] Bao Y, Wang J, He F, Ma H, Wang H (2012) Molecular cytogenetic identification of a wheat (*Triticum aestivum*)-American dune grass (*Leymus mollis*) translocation line resistant to stripe rust. Genet Mol Res 11: 3198–3206. 10.4238/2012.May.22.2 22653669

[18] Pang YH, Chen XH, Zhao JX, Du WL, Cheng XN, Wu J, et al (2014) Molecular cytogenetic characterization of a wheat—*Leymus mollis* 3D(3Ns) substitution line with resistance to leaf rust. J Genet Genomics 41: 205–214. 10.1016/j.jgg.2013.11.008 24780618

[19] Wang XP, Fu J, Zhang XQ, Jing JK, Wen YX (2000) Molecular cytogenetic study on genome constitutions of octoploid *Tritileymus* . Acta Botanica Sinica 42: 582–586.

[20] Zhao JX, Du WL, Wu J, Cheng XN, Gao Y, Pang YH, et al (2012) Development and identification of a wheat-*Leymus mollis* multiple alien substitution line. Euphytica 190: 45–52.

[21] Yang XF, Wang CY, Chen CH, Zhang H, Tian ZR, Li X, et al (2014) Chromosome constitution and origin analysis in three derivatives of Triticum aestivum—*Leymus mollis* by molecular cytogenetic identification. Genome 57: 583–591. 10.1139/gen-2014-0161 25760775

[22] Jiang J, Friebe B, Gill B (1994) Recent advances in alien gene transfer in wheat. Euphytica 73: 199–212.

[23] Schwarzacher T, Anamthawat-Jonsson K, Harrison GE, Islam AKMR, Jia JZ, King IP, et al (1992) Genomic *in situ* hybridization to identify alien chromosomes and chromosome segments in wheat. Theor Appl Genet 84: 778–786. 10.1007/BF00227384 24201474

[24] Zhang P, Friebe B, Gill BS, Park RF (2007) Cytogenetics in the age of molecular genetics. Aust J Agr Res 58: 498–506.

[25] Han FP, Liu B, Fedak G, Liu Z (2004) Genomic constitution and variation in five partial amphiploids of wheat-*Thinopyrum intermedium* as revealed by GISH, multicolor GISH and seed storage protein analysis. Theor Appl Genet 109: 1070–1076. 15197444 10.1007/s00122-004-1720-y

[26] Doyle JJ, Doyle JL (1987) A rapid DNA isolation procedure for small quantities of fresh leaf tissue. Phytochem Bull 19: 11–15.

[27] Tang ZX, Yang ZJ, Fu SL (2014) Oligonucleotides replacing the roles of repetitive sequences pAs1, pSc119.2, pTa-535, pTa71, CCS1, and pAWRC.1 for FISH analysis. J Appl Genet 55: 313–318. 10.1007/s13353-014-0215-z 24782110

[28] Hu LJ, Li GR, Zeng ZX, Chang ZJ, Liu C, Zhou JP, et al (2010) Molecular cytogenetic identification of a new wheat-*Thinopyrum* substitution line with stripe rust resistance. Euphytica 177: 169–177.

[29] Ishikawa G, Nakamura T, Ashida T, Saito M, Nasuda S, Endo TR, et al (2009) Localization of anchor loci representing five hundred annotated rice genes to wheat chromosomes using PLUG markers. Theor Appl Genet 118: 499–514. 10.1007/s00122-008-0916-y 19057889

[30] Ma H, Singh RP, Mujeeb-Kazi A (1995) Suppression/expression of resistance to stripe rust in synthetic hexaploid wheat (*Triticum turgidum* × *T. tauschii*). Euphytica 83: 87–93.

[31] Wang J, Chen XH, Du WL, Zhao JX, Wu J, Cheng XN, et al (2012) Morphological and molecular cytogenetic characterization of partial octoploid *Tritileymus* . Genet Resour Crop Ev 60: 1453–1462.

[32] Rayburn AL, Gill BS (1986) Isolation of a D-genome specific repeated DNA sequence from *Aegilops squarrosa* . Plant Mol Biol Rep 4: 102–109.

[33] Leitch IJ, Heslop-Harrison JS (1992) Physical mapping of the 18S-5.8 S-26S rRNA genes in barley by *in situ* hybridization. Genome 35: 1013–1018.

[34] Uhrin A, Szakács É, Láng L, Bedő Z, Molnár-Láng M (2011) Molecular cytogenetic characterization and SSR marker analysis of a leaf rust resistant wheat line carrying a 6G(6B) substitution from *Triticum timopheevii* (Zhuk.). Euphytica 186: 45–55.

[35] Lei M, Li G, Zhang S, Liu C, Yang Z (2011) Molecular cytogenetic characterization of a new wheat *Secale africanum* 2R^a^ (2D) substitution line for resistance to stripe rust. J Genet 90: 283–287. 21869476 10.1007/s12041-011-0081-y

[36] Du WL, Zhao JX, Wang J, Wang LM, Wu J, Yang QH, et al (2014) Cytogenetic and Molecular Marker-Based Characterization of a Wheat-*Psathyrostachys huashanica* Keng 2Ns(2D) Substitution Line. Plant Mol Biol Rep: 1–10.

[37] Li GR, Zhao JM, Li DH, Yang EN, Huang YF, Liu C, et al (2014) A novel wheat-*Dasypyrum breviaristatum* substitution line with stripe rust resistance. Cytogenet Genome Res 143: 280–287. 10.1159/000366051 25247402

[38] Heslop-Harrison JS (2000) Comparative genome organization in plants: from sequence and markers to chromatin and chromosomes. Plant Cell 12: 617–636. 10810139 10.1105/tpc.12.5.617 PMC139916

[39] Kong F, Wang H, Cao A, Qin B, Ji J, Wang SL, et al (2008) Characterization of *T*. *aestivum*-*H*. *californicum* chromosome addition lines DA2H and MA5H. J Genet Genomics 35: 673–678. 10.1016/S1673-8527(08)60089-2 19022201

[40] Wu J, Yang XM, Wang H, Li HJ, Li LH, Li XQ, et al (2006) The introgression of chromosome 6P specifying for increased numbers of florets and kernels from *Agropyron cristatum* into wheat. Theor Appl Genet 114: 13–20. 17031609 10.1007/s00122-006-0405-0

[41] Qi LL, Pumphrey MO, Friebe B, Zhang P, Qian C, Bowden RL, et al (2011) A novel Robertsonian translocation event leads to transfer of a stem rust resistance gene (Sr52) effective against race Ug99 from Dasypyrum villosum into bread wheat. Theor Appl Genet 123: 159–167. 10.1007/s00122-011-1574-z 21437597

[42] Said M, Cabrera A (2009) A physical map of chromosome 4Hch from *H. chilense* containing SSR, STS and EST-SSR molecular markers. Euphytica 167: 253–259.

[43] Wang D, Zhuang LF, Sun L, Feng YG, Pei ZY, Qi ZJ (2010) Allocation of a powdery mildew resistance locus to the chromosome arm 6RL of *Secale cereale* L. cv. ‘Jingzhouheimai’. Euphytica 176: 157–166.

[44] Chen GL, Zheng Q, Bao YG, Liu SB, Wang HG, Li XF (2012) Molecular cytogenetic identification of a novel dwarf wheat line with introgressed *Thinopyrum ponticum* chromatin. J Biosciences 37: 149–155. 10.1007/s12038-011-9175-1 22357212

[45] Du WL, Wang J, Wang LM, Zhang J, Chen XH, Zhao JX, et al (2013) Development and characterization of a *Psathyrostachys huashanica* Keng 7Ns chromosome addition line with leaf rust resistance. PloS one 8: e70879 10.1371/journal.pone.0070879 23976963 PMC3747159

[46] Hu LJ, Li GR, Zhan HX, Liu CG, Yang ZJ (2012) New St-chromosome specific molecular markers for identifying wheat-*Thinopyrum intermedium* derivative lines J Genet 91: e69–e74. 22932422

